# Bioactive Polysaccharides from Seaweeds

**DOI:** 10.3390/molecules25143152

**Published:** 2020-07-09

**Authors:** Faiez Hentati, Latifa Tounsi, Djomdi Djomdi, Guillaume Pierre, Cédric Delattre, Alina Violeta Ursu, Imen Fendri, Slim Abdelkafi, Philippe Michaud

**Affiliations:** 1 Université Clermont Auvergne, CNRS, SIGMA Clermont, Institut Pascal, F-63000 Clermont-Ferrand, France; faizhentati@gmail.com (F.H.); latifa.tounsi@enis.tn (L.T.); guillaume.pierre@uca.fr (G.P.); cedric.delattre@uca.fr (C.D.); alina-violeta.ursu@sigma-clermont.fr (A.V.U.); 2 Laboratoire de Génie Enzymatique et Microbiologie, Équipe de Biotechnologie des Algues, Département Génie Biologique, Ecole Nationale d’Ingénieurs de Sfax, Université de Sfax, Sfax 3038, Tunisie; slim.abdelkafi@enis.tn; 3 Department of Renewable Energy, National Advanced School of Engineering of Maroua, University of Maroua, P.O. Box 46 Maroua, Cameroon; ngdjomdi@yahoo.fr; 4 Institut Universitaire de France (IUF), 1 rue Descartes, 75005 Paris, France; 5 Laboratoire de Biotechnologie des Plantes Appliquée à l’Amélioration des Cultures, Faculté des Sciences de Sfax, Université de Sfax, Sfax 3038, Tunisie; imen.fendri@fss.usf.tn

**Keywords:** Macroalgae, seaweeds, biomolecules, polysaccharides, bioactive agents

## Abstract

Bioactive compounds with diverse chemical structures play a significant role in disease prevention and maintenance of physiological functions. Due to the increase in industrial demand for new biosourced molecules, several types of biomasses are being exploited for the identification of bioactive metabolites and techno-functional biomolecules that are suitable for the subsequent uses in cosmetic, food and pharmaceutical fields. Among the various biomasses available, macroalgae are gaining popularity because of their potential nutraceutical and health benefits. Such health effects are delivered by specific diterpenes, pigments (fucoxanthin, phycocyanin, and carotenoids), bioactive peptides and polysaccharides. Abundant and recent studies have identified valuable biological activities of native algae polysaccharides, but also of their derivatives, including oligosaccharides and (bio)chemically modified polysaccharides. However, only a few of them can be industrially developed and open up new markets of active molecules, extracts or ingredients. In this respect, the health and nutraceutical claims associated with marine algal bioactive polysaccharides are summarized and comprehensively discussed in this review.

## 1. Introduction

Promising biologically active compounds isolated from natural sources have revealed proven activities in the cosmetic, medical and pharmaceutical fields. Several industries consider the marine ecosystem as a source of natural compounds with multiple activities. In it, marine macroalgae (Chlorophyceae, Pheophyceae and Rhodophyceae) constitute the richest source of non-animal biological compounds in nature [1].

The global production of macroalgae generated a turnover of around 10 billion US dollars for 28–30 million tons (Mt) of fresh algae collected or cultivated in 2016 [2]. The seaweed market is more mature and substantial in volume than the microalgae one. China, Indonesia and the Philippines dominate the algal world production with respective levels of 48.7, 36.6 and 5.7% [2,3,4].

Furthermore, this production only concerns red (approximately 60%) and brown (approximately 39.5%) algae. Currently, 95% of the world tonnage comes from seaweed farming (with a growth rate of 5.5%/year) compared to only 5% from the harvest of wild algae (1.5 Mt) [5]. Approximately, at least 291 species from 43 countries are used in the world, and they are divided into 163 species of Rhodophyceae, 75 of Pheophyceae and 33 of Chlorophyceae [3]. Nonetheless, six species of macroalgae represent 96% of the world production volume, i.e., *Eucheuma* (9.55 Mt), *Laminaria* (7.65 Mt), *Gracilaria* (3.75 Mt), *Undaria* (2.36 Mt), *Porphyra* (1.80 Mt) and *Kappaphycus* (1.64 Mt) [3,5].

Seaweeds are known as producers of various biologically active macromolecules (polyphenols, diterpenes, fiber, proteins and notably polysaccharides) with different structural and physicochemical properties and interesting functional characteristics. Regarding the production of these bioactive substances, algae are considered the most abundant source of polysaccharides, which may be sulfated (fucoidans, carrageenans, galactans, and agars) and non-sulfated (alginates, laminaran) [6]. These high molar mass biopolymers (10 to 1000 kDa) have extremely varied structures and are mainly made up of osidic unit sequences (pentoses and/or hexoses) linked by *O*-glycosidic bonds. The repeating unit can be composed of the same monosaccharide (homopolysaccharides) or of different units (heteropolysaccharides). The monosaccharide sequences can consist of neutral ones (e.g., Glc, Gal, Xyl, Fuc, Ara, Man), acidic ones (GlcA, GalA, etc.) or hexosamines (GlcNAc, GalNAc) [1,6]. It was reported that these polymers can be linear (alginates, cellulose), but also branched (fucoidans, sulfated galactans). They can be substituted by proteins but also by organic groups such as acetate, lactate, pyruvate and succinate or inorganic groups like phosphate, sulfate and amine. In this case, they are called aglycones [1]. This review focuses on marine seaweed polysaccharides and gives a recent overview of their structural, physicochemical and biological features with potential health benefits.

## 2. Marine-Algal Bioactive Compounds

**Figure 1:**
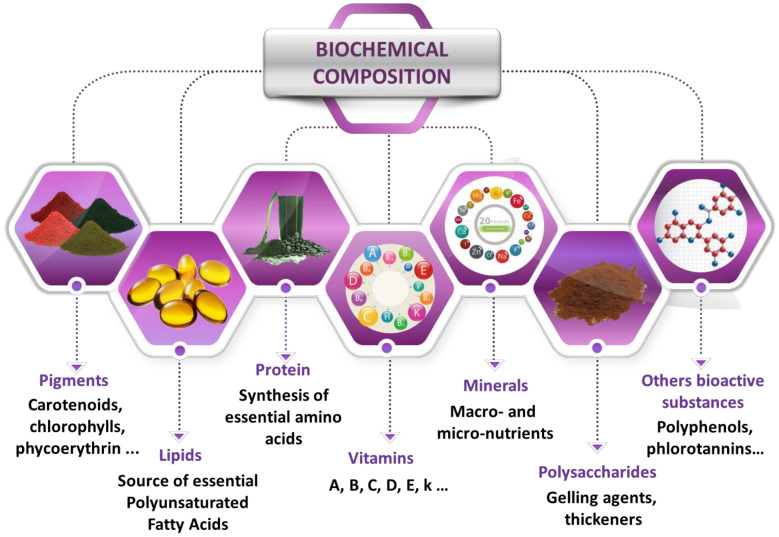
Bioactive compounds from marine seaweeds.

Marine seaweeds are considered as an excellent source of high-value biologically active molecules (functional substances) thanks to their abundance and the advantage of their environmentally friendly cultivation processes [7,8]. Setting optimal extraction conditions of bioactive compounds from the seaweed matrix is of crucial importance for obtaining homogeneous biological activities and subsequent industrial applications. Seaweeds, or their extracts, are rich in vital nutrients and can produce a great variety of bioactive compounds. Among them, polyphenolic constituents, terpenoids, carotenoids, vitamins, phlorotannins, alkaloids, diterpenes tocopherols, tocotrienols, proteins, peptides and carbohydrates (polysaccharides) are of great interest [9] (Figure 1).

These bioactive compounds display several nutraceutical effects, such as anticoagulant, immunomodulatory, anticancer, antitumor, antioxidant, antiallergic, anti-inflammatory, hypoglycemic, antiobesity, antimicrobial, antifungal, and antiviral activities [7,10,11].

The use of conventional extraction techniques negatively affects the biomolecule production yield as well as its bioactivity [12,13]. Hence, it is necessary to develop new, proficient and innovative extraction procedures with remarkable advantages over the conventional technologies to obtain high quality biomolecules with a greater yield. However, using more environmental, efficient and economic processes based on the green extraction concept has allowed us to develop new non-conventional technologies to recover valuable compounds from algae biomass [12,14,15]. Some of these novel techniques are microwave assisted extraction (MAE), pressurized fluid/liquid extraction (PLE), ultrasound-assisted extraction (UAE), supercritical fluid extraction (SFE) and enzyme-assisted extraction (EAE), which are also used in pharmaceutical and food industries [16,17,18].

### 2.1. Pigments and Phenolic Compounds

Pigments present in marine seaweeds are divided into three types: chlorophylls, carotenoids and phycobiliproteins. Chlorophylls are greenish fat-soluble pigments which play a key role in the photosynthesis phenomenon and are commonly found in land plants, algae, and cyanobacteria [19]. The main algae carotenoids include carotenes, fucoxanthin (the most abundant carotenoids), lycopene, astaxanthin, zeaxanthin, neoxanthin, lutein and violaxanthin [19]. Phycobiliproteins are water-soluble pigments, distinguishing three types of molecules with different protein structures. Phycoerythrins are red pigments (the most abundant ones)) whereas phycocyanins and allophycocyanins are respectively blue and light blue pigments [19]. These pigments have substantial potentials as biologically active agents, nutraceutical ingredients and food colorings with anticancer, anti-inflammatory, antidiabetic, immunomodulatory, antioxidant and antiangiogenic properties [19].

Phenolic compounds from marine algae include phenolic acids, phlorotannins, flavonoids, tannins and catechins. The type and yield extraction of phenolic compounds strongly depend on the seaweed species. In fact, brown seaweeds (Pheophyceae) are mainly characterized by significant levels of phlorotannins, complex polymers composed of oligomers of phloroglucinol (1,3,5-trihydroxybenzene), whereas red (Rhodophyceae) and green (Chlorophyceae) seaweeds are rich in flavonoids, phenolic acids and bromophenols [20]. Numerous biological activities have been ascribed to polyphenols isolated from seaweeds such as antitumor, anticancer, antimicrobial, antiviral, antiobesity, antiproliferative, anti-inflammatory, antidiabetic and antioxidant properties [20]. Ryu et al. [21] and Gullón et al. [8] proved the in vitro anti-inflammatory property of a polyphenol-rich fraction isolated from Rhodophyceae. In addition, Liu et al. [22,23] and Gullón et al. [8] demonstrated that phlorotannins and bromophenols extracted from red and green algae have a great inhibition activity against in vitro cancer cell proliferation and in vivo tumor growth, along with in vitro antidiabetic and antithrombotic activities.

### 2.2. Lipids and Proteins

Lipid or fatty acid content of marine seaweeds varies according to many factors, such as geographical location, season, climatic conditions (temperature, light intensity), salinity and algal species. Usually, algae have a low lipid content lower than 5.0% of their dry weight (*w/w*) [24] (Table 1). The fatty acid profile of algae contains wide quantities of polyunsaturated fatty acids (PUFAs), such as docosahexaenoic (DHA, C22:6 n-3), eicosapentaenoic (EPA, C20:5 n-3), α-linolenic (ALA, C18:3 n-3), linoleic (LA, C18:2 n-6), octadecatetraenoic (SDA, C18:4 n-3), and arachidonic (AA, C20:4 n-6) acids [24,25,26]. Sterols mainly represented by fucosterol, clionasterol, isofucosterol and cholesterol are the main nutritional constituents of marine seaweeds [26,27,28]. Sterols have important nutritional and biological properties, such as anticancer, antioxidant, antiobesity, antitumoral, antiviral, and are effective against cardiovascular diseases [24].

Proteins, peptides and amino acids content in seaweeds ranges from 5% to 47% of their dry weight (*w/w*) depending on many factors, such as algal species, season and geographical location [7] (Table 1). The red seaweeds *Porphyra tenera* and *Palmaria palmata* possess high protein concentrations of 35% (*w*/*w*) and 47% (*w*/*w*), respectively, whereas the green alga *Ulva pertuse* has a protein content of only 26% (*w*/*w*) [29]. Generally, Chlorophyceae and Rhodophyceae have higher protein contents compared to Pheophyceae [7,30]. Besides, seaweed proteins are excellent sources of most amino acids, such as proline, alanine, glycine, arginine, and especially aspartic and glutamic acids [7,30,31]. Peptides exhibiting a large spectrum of bioactivities can be obtained from the protein fraction extracted from marine seaweeds. Moreover, phycobiliproteins, intensely fluorescent proteins isolated from the red alga *P. palmata*, could be used in the prevention of hypertension due to their great angiotensin-converting enzyme (ACE) inhibitory activity [32].

### 2.3. Vitamins and Minerals

Seaweeds are important sources of hydro-and liposoluble vitamins, which could improve the food and feed vitamin status. They consist of water-soluble vitamins B (B1, B2, B3, B6, B12), C, niacin, folic acid, pantothenic acid and riboflavin, as well as fat-soluble vitamins A, D, E, and carotenoids as provitamin forms of vitamin A [7,51,52]. For instance, the values mentioned for vitamin C were in a similar range for green, red, and brown seaweeds (0.0347–1.25, 0.0353–1.61, 0.0345–1.85 g/100 g dry weight (DW), respectively) [8]. However, the literature data concerning vitamin B12 content are more scattered, ranging between 0.06 and 0.786 g/100 g DW for green seaweeds, 0.0961 and 1.34 g/100 g DW for red seaweeds and from 0.0164 to 0.0431 g/100 g DW for brown seaweeds [8,53]. Furthermore, important vitamin B3 values are recorded in the range of 0.005–1.0 g/100 g DW for Chlorophyceae, 0.0951-0.10 g/100 g DW for Rhodophyceae and 0.612–0.90 g/100g DW for Pheophyceae [8].

Macroalgae are also wealthy sources of minerals. Their mineral amount ranges between 7 to 40% of their dry weight (*w*/*w*) according to factors such as algal species, season and geographical collection site [8,54,55]. Seaweeds have a significant amount of macroelements Ca, K, P, Na, Mg, Mn, Fe and trace elements (microelements) Pb, Cu, Zn, Sc, Sd, As, Sr and Cr [8,56]. These elements—especially calcium (Ca)—are found in seaweeds with higher levels than in terrestrial plants [56]. The important iodine levels found in algal biomass differ from species and range from 0.004 to 2.66 g/kg [14]. However, it is worthwhile to note that new strategies have been applied to reduce its content in macroalgae food products because of its potential harmful properties for health [8].

### 2.4. Carbohydrates

Marine macroalgae are considered as good sources of carbohydrates varying in its total content from 5 to75% (*w/w*, DW) depending on the age, species, period and harvesting site [7,11] (Table 1). Algal carbohydrates consist mainly of polysaccharides and few amounts of disaccharides and monosaccharides [1]. Polysaccharides isolated from marine seaweeds are found principally in sulfated and non-sulfated forms [8]. The presence of various types of polysaccharides (matrix and storage ones) is macroalgae species-specific. For example, green marine seaweeds are rich in ulvans, brown macroalgae contain alginic acids (or alginates), laminarans (or laminarins) and fucoidans, whereas red seaweeds are characterized by their carrageenans, agars, xylogalactans (especially in the Corallinales order), sulphated galactans, xylans, porphyran and floridean starch [8,11,57,58]. Abundant and recent investigations have widely described valuable biological activities of native seaweed polysaccharides but also of their derivatives, including oligosaccharides and (bio)chemically modified polysaccharides. Among these activities, anti-inflammatory [59], antidiabetic [60], antiobesity [61], antihyperlipidemic [62], immunomodulatory, antioxidant [11,59], antitumor [63], antiviral, antimicrobial [64], and gastroprotective [65] activities have been well explored. Thus, the biological properties of marine algal bioactive polysaccharides are summarized and comprehensively discussed later in this review.

## 3. Main Structural Features of Algal Polysaccharides

**Figure 2:**
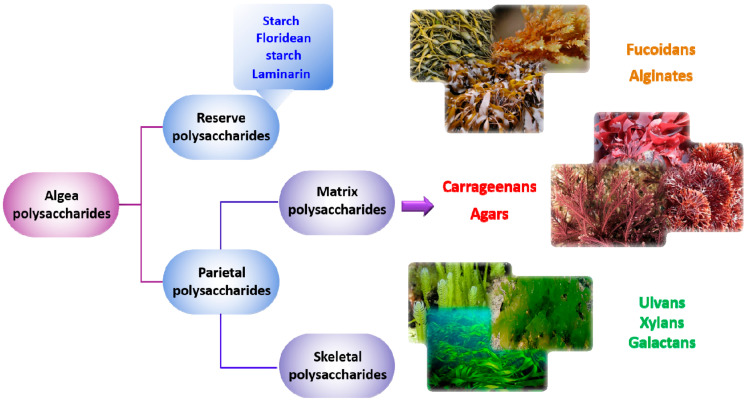
Algal polysaccharides classification.

The algal walls are distinguished from those of terrestrial plants by the predominance of mucilage on the skeleton, the abundance of sulfated macromolecules of polyanionic character on neutrals and the presence of an abundant intercellular matrix [1]. All of these characteristics give particular properties to algal cells, such as (i) mechanical resistance to deformation of the thalli (ensured by celluloses, alginates and galactans), (ii) improving elasticity and rigidity of these networks by incorporating CaCO_3_ associated with MgCO_3_ and strontium (Sr), (iii) the increase in ionic exchanges by capturing cations such as Mg^2+^, Ca^2+^ or Na^+^ and, finally, (iv) the strong adaptation against dehydration by the presence of sulfated polysaccharides [66]. The cell walls of algae are composed of two parts: (*i*) a so-called “crystalline” phase which plays the role of skeleton and (ii) an amorphous phase, called the “matrix”, which contains the skeleton. For each of the three classes of macroalgae, the crystalline phase consists of cellulose (β-(1→4)-d-Glc*p*) and shows little variation from green to brown algae [1] (Figure 2).

The storage (reserve) polysaccharides result directly from the photosynthetic mechanism. They are stored in algae plastids and are reusable on demand to maintain the basic metabolism. In green and red seaweeds, polysaccharides are α-(1→4) and α-(1→6)-d-glucans with comparable structures to starch of terrestrial plants, while β-(1→3)-d-glucans (laminarin) is found in brown algae [1]. On the other hand, the matrix polysaccharides of the amorphous phase are very different from one algal class to another, and those of brown and red algae have many industrial applications as food texturizers.

**Figure 3:**
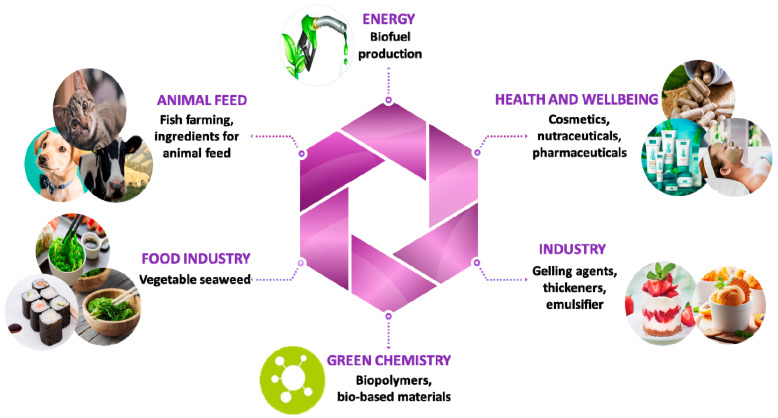
Applications of bioactive algal polysaccharides.

These phycocolloids, also known as hydrocolloids, are substances capable of modifying the rheological properties of the aqueous solutions which contain them. They can thus modify the flow properties of water, being classified as thickeners (changes in water mobility), gelling agents (stopping water mobility) and finally stabilizers (limiting flocculation, flotation, decantation or coalescence of particles in a liquid medium). Algal polysaccharides are commonly used as texturing agents in the food, pharmaceutical and cosmetic industries [57] (Figure 3).

These biological activities and rheological properties are strongly influenced by their structures (monosaccharidic composition, anomeries, glycosidic bonds and branching degree) and their molar masses [67]. Note that understanding the precise functions of polysaccharides in different biological mechanisms requires the development of efficient separation and purification techniques, which will allow one to study their structure–function relationships.

### 3.1. Brown Seaweed Polysaccharides

**Figure 4:**
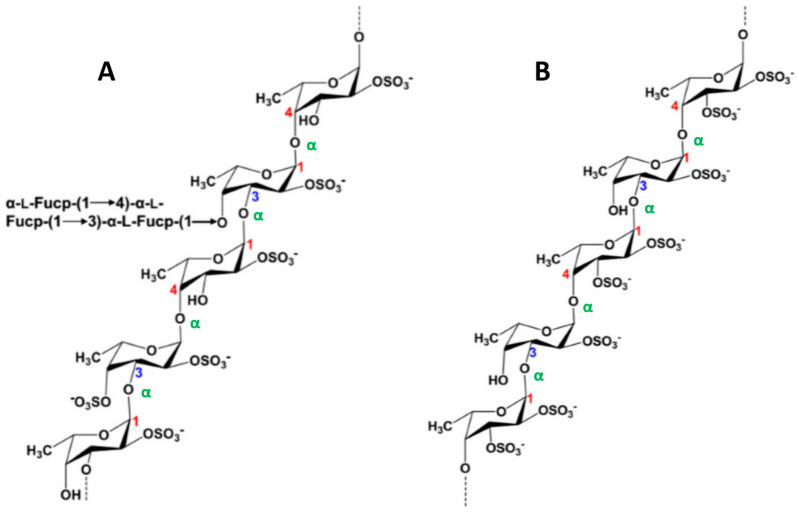
Structures of fucoidans extracted from brown seaweeds of the Fucales order. (**A**) Fucoidan of *Fucus serratus* (and *Ascophyllum nodosum*) composed of a main chain of (1→3)- and (1→4)-α-l-Fuc*p* with short branches of α-l-Fuc*p*-(1→4)-α-l-Fuc*p* and α-l-Fuc*p*-(1→3)-α-l-Fuc*p* in *O*-4 of α-(1→3)-l-Fuc*p* and sulfate groups in *O*-2 and/or *O*-4 positions. (**B**) Fucoidan extracted from *Fucus evanescens* consisting of a main skeleton of (1→3)- and (1→4)-α-l-Fuc*p* highly substituted by sulfate groups at *O*-2 and/or *O*-3 positions.

**Figure 5:**
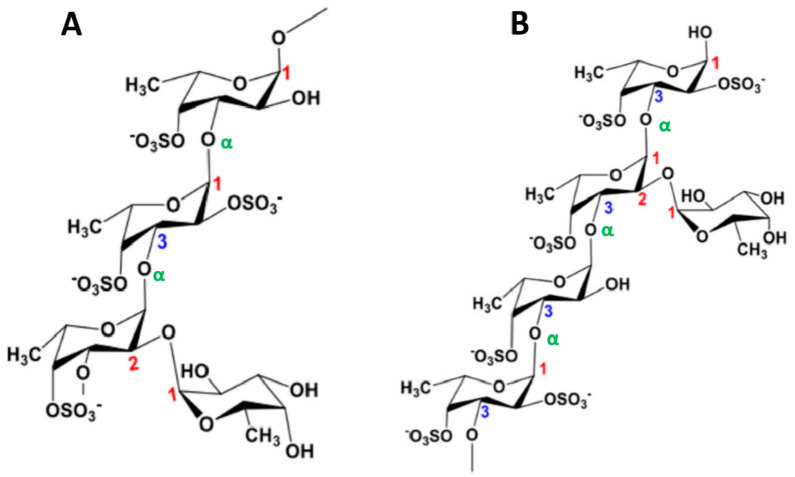
Structures of fucoidans from brown seaweeds of the Laminariales order. (**A**) Fucoidan from *Laminaria saccharina* composed of a main chain of (1→3)-α-l-Fuc*p* branched at *O*-2 and *O*-4 of α-l-Fuc*p* by terminal residues and sulfate groups. (**B**) Fucoidan obtained from *Chorda filum* consisting of a (1→3)-α-l-Fuc*p* main backbone highly ramified at *O*-2 by terminal residues and substituted by sulfate groups at *O*-2 and/or *O*-4positions.

In agreement with IUPAC recommendations, Berteau and Mulloy [68] defined sulfated fucans polysaccharides from marine invertebrates (notably sea cucumbers and sea urchin eggs) principally composed of l-fucose residues (less than 10% of other monosaccharides) and fucoidan for sulfated fucans isolated from marine seaweeds. Complex fucoidans, extracted from macroalgae, are thus sulfated and branched α-l-fucans containing predominantly sulfated l-Fuc*p* (< 90%), but also other monosaccharides such as d-Gal*p*, d-Man*p* and d-Xyl*p*, as well as uronic acids (d-GlcA*p* and sometimes d-GalA*p*) [11,68]. The complexity and structural heterogeneity of fucoidans vary with the extraction processes and algal sources, but also with the local climatic conditions and harvest site [11]. Several studies have attempted to determine the exact structure of fucoidans, and only a few examples of regular patterns have been described. The type of *O*-glycosidic bonds, the position of the sulfates and the ramifications seem to be variable [69]. It has been reported by Bilan et al. [70], Sellimi et al. [71] and Hentati et al. [11] that representatives of the Fucales order (e.g., *Fucus*, *Sargassum*, *Pelvetia*, *Ascophyllum* and *Cystoseira*) (Figure 4) contain fucoidans with a main backbone of (1→2), (1→3) and (1→4)-α-l-Fuc*p*, while fucoidans obtained from the Laminariales order (e.g., *Laminaria*, *Ecklonia* and *Eisenia*) (Figure 5) have a linear chain of α-l-fucopyranosyl residues linked in α-(1→3) [72]. The sulfate and/or acetate groups of the α-l-Fuc*p* residues are predominately situated in position C-2 [73], C-4 [11] and occasionally in C-3 [74], or disubstituted at positions C-2 and C-4 [70].

These sulfated polysaccharides have a broad spectrum of biological activity, including antioxidant, anticoagulant, antithrombotic, antiproliferative, antitumor, anticancer, immunomodulatory, anti-inflammatory, antibacterial and antidiabetic activities [7].

Ascophyllans, called xylofucoglucuronanes, are more complex and composed of a poly-(1→4)-β-d-glucuronan skeleton, branched by short chains containing d-Xyl*p* and l-Fuc*p* sulfated in position C-4 [75]. In contrast, sargassans (glucuronofucogalactans) are similar to the previous ones, but also contain d-Man*p* residues. This type of polysaccharide has been identified in particular in the genus *Sargassum* (e.g., *Sargassum linifolium*) [76]. Generally, fucoidan solutions displayed a low shear-thinning behavior (sometimes Newtonien) with low viscoelastic characteristics weakly influenced by monovalent and divalent salts [77].

**Figure 6:**
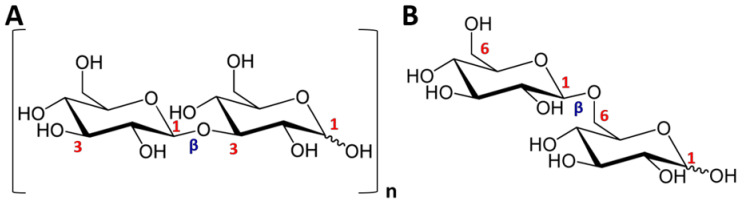
Structures of (**A**) laminaribioses and (**B**) gentiobioses.

Laminarin (named also laminaran) from brown seaweeds is an original reserve polysaccharide isolated initially from the large *laminaria* present in the North Atlantic. Laminarin, with a Dp of 15-40 and therefore an M_w_ of 2-10 kDa, is a β-(1→3)-d-glucan whose diholosidic repeating unit is laminaribiosis (Figure 6).

The laminarin chains have ramifications consisting of β-(1→6)-d-Glc*p*, which is verified by obtaining gentiobiose after hydrolysis [1]. Two types of laminarin have been described, namely (i) the M series (or M-chains) where the laminarin chains carry a d-mannitol residue at their reducing terminal end and (ii) the G series (or G-chains) where the d-mannitol residue is replaced by d-Glc*p* unit. Depending on the percent of M chains in a total laminarin structure, a distinction can be made between soluble laminarin (around 75% of M-chains) and insoluble laminarin (M-chains ≤ 45%). Laminarins have low cell toxicity, proven biodegradability and high biocompatibility. Diverse ranges of biological activities have been reported for this polysaccharide. Among them, we can mention antiapoptotic, anti-inflammatory, immunoregulatory, antitumor, anticoagulant and antioxidant activities [78]. Brown algae also use mannitol and laminitol as energy stores. These carbohydrates are abundant in the fronds and rarely present in the stipes.

**Figure 7:**
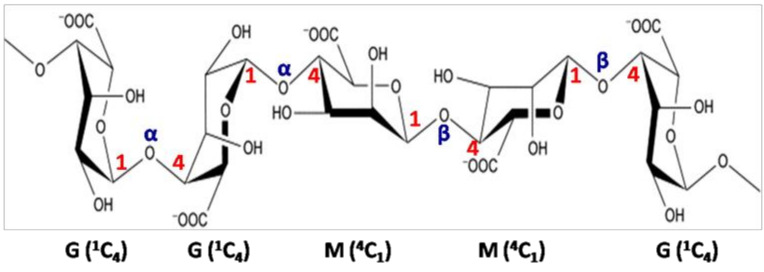
Structure of alginates. G: guluronate or l-Gul*p*A; M: mannuronate or d-Man*p*A.

With 12,863 research articles, 411 reviews and 15,322 patents, the term alginate(s) is used to designate alginic acids and their salt forms which constitute a group of unbranched polyuronides [79]. They are mainly derived from the cell walls of brown seaweeds and some of them are produced as exopolysaccharides (acetylated at C-2 and/or C-3 of M residues) by some *Pseudomonas* and *Azetobacter* species [80]. Their existence in cell walls of Pheophyceae in different salt forms (Ca^2+^, Na^+^ or Mg^2+^) makes the tissues strong and more flexible. These polysaccharides are composed of (1→4)-β-d-mannuronic acid (d-Man*p*A) with a ^4^C_1_ ring conformation and α-l-guluronic acid (l-Gul*p*A) with a ^1^C_4_ ring conformation (Figure 7). They are arranged in homogeneous (MM (poly-M) or GG (poly-G)) and heterogeneous (MG or GM (poly-MG)) blocks leading to a large diversity of structures, molar masses, physicochemical and biological properties (Figure 7) [11,79].

Fundamentally, alginates are characterized by their molecular weights (M_w_, M_n_, Dp), polydispersity index (PI = M_w_/M_n_), macromolecular parameters (e.g., intrinsic viscosity ([η]), critical concentration (C*), hydrodynamic (R_h_) and gyration (R_g_) radii), as well as notably by M/G ratio and number and length of monad (M and G) and diad (GG, MM, MG or GM) frequencies, which provide structural information that is easily correlated with their rheological properties (gelling or/and thickening) in a solution or in the presence of mono- and di-valent salts [11,77,81]. Indeed, M block segments offer linearity and flexibility to a linear chain of alginates, whereas G blocks provide rigid and folded structural conformations responsible for the stiffness of these anionic copolymers. Furthermore, GG-rich alginates have higher viscosity (pseudoplastic/shear-thinning behavior) with important viscoelastic properties. The gelling properties of alginate are also related to their GG blocks where selective alkaline earth metal multivalent (divalent (whose the most used is Ca^2+^) or trivalent) cations take place by chelation, leading to the formation of stronger gels described by the egg-box model. Alginates with M/G > 1 are more suitable to form elastic gels than brittle ones [11,77,79,82]. Alginates are employed mainly in the feed, food, cosmetic and pharmaceutical industries for their thickening and gelifying characters.

### 3.2. Red Seaweed Polysaccharides

Red seaweed (Rhodophyta) is a known industrial source of water-soluble sulfated galactans with gelling and texturing properties such as (i) carrageenans and (ii) agarocolloids. These two polymers have similar structures and are constructed on the basis of a linear chain consisting of alternating (1→3)-β-Gal*p* and (1→4)-α-Gal*p* units [57]. The β-Gal*p* unit (A unit) always belongs to the d series, while the α-Gal*p* unit (B unit) is of configuration d in carrageenans and l in agarocolloids (agarans). The (1→4)-linked α-Gal*p* units can exist in the 3,6-anhydrogalactose form (3,6-α-AnGal*p*, DA units). The latter form is obtained by elimination of the sulfate ester at C-6 of the (1→4)-linked α-Gal*p* unit in the presence of galactose-6-sulfurylases during biosynthesis [83].

**Figure 8:**
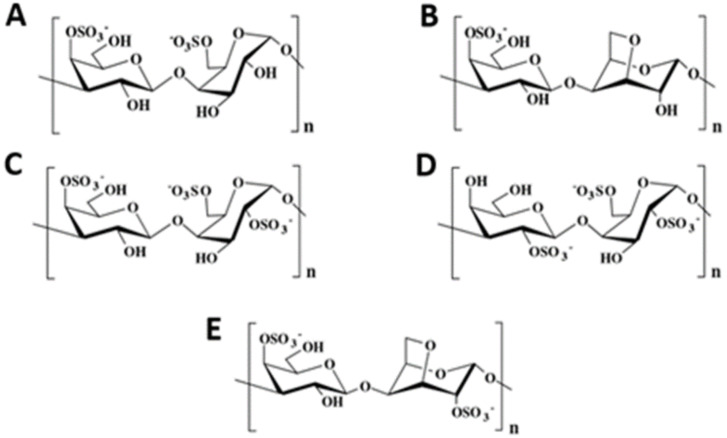
Structures of (**A**) μ-carrageenans, (**B**) κ-carrageenans, (**C**) ν-carrageenans, (**D**) λ-carrageenans and (**E**) ι-carrageenans.

Carrageenan is the third-largest hydrocolloid in the food industry, after gelatin and starch. Sulfated galactans with B residues of the d-series (4-α-d-Gal*p*) are named carrageenans [84]. These are linear polymers of carrabiosis (sulfated dioside), which alternately consists of repeating AB units (3-β-d-Gal*p* and 4-α-d-Gal*p*). The B residues (α-d-Gal*p*) can be replaced by 3,6-α-d-AnGal*p*. Carrageenans can be extracted from diverse species of Rhodophyceae, such as *Agardhiella*, *Chondrus*, *Eucheuma*, *Furcellaria*, *Gigartina*, and *Hypnea* [85,86]. Their structural heterogeneity depends on their levels of sulfate substituents and 3,6-α-d-AnGal*p* residues, as well as their solubility in potassium chloride (KCl) [85]. Besides, the carrageenan’s main skeleton may contain other monosaccharides (Xyl*p*, Glc*p*, GlcA*p* and GalA*p*), pyruvic acid ketals and methyl ether groups [57]. According to many authors, these sulfated polysaccharides are divided into several structural families (κ, λ, β, ω) where the kappa (κ), iota (ι), and lambda (λ) are the well-known structural groups of carrageenans [87] (Figure 8). It is important to note that the molecular weights of carrageenans are very variable, ranging from 200 to 1400 kDa [85,87]. The sulfate binding sites are always on carbons 2, 4 and 6 for the two units A and B (or DA). κ-,ι-,λ-carrageenans respectively contain one, two and three sulfates per disaccharide unit, which corresponds to sulfation rates of around 20, 33 and 41%. The kappa family includes the κ- (A4S-DA) and ι- (A4S-DA2S) carrageenans and their respective precursors, μ- (A4S-B6S) and ν- (A4S-B2S, 6S). The lambda family consists of sulfated carrageenans such as λ- (A2S-B2S,6S), θ- (A2S-DA2S), ξ- (A2S-B2S) and pyruvilated structures such as π-carrageenan (A2S-BP2S). The beta family have non-sulfated A units for the β- (A-DA) and α- (A-DA2S) carrageenans and their respective precursors, γ- (A-B6S) and δ- (A-B2S,6S). Finally, the omega family includes ω-carrageenan (A6S-DA) and its precursor ψ-carrageenan (A6S-B6S).

**Figure 9:**
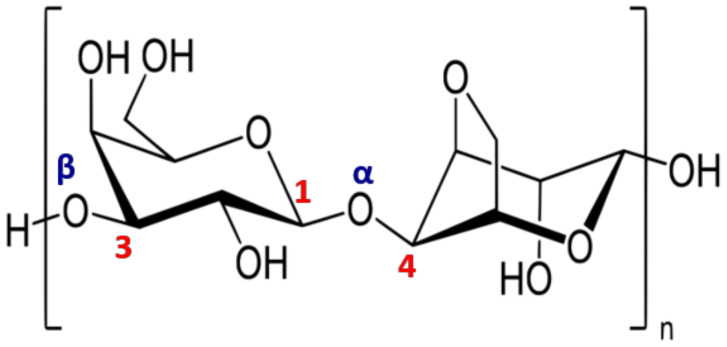
Representation of agarose.

Sulfated galactans with B residues of the l-series (4-α-l-Gal*p*) are named agarans [84]. Agarans are divided in two groups, i.e., agars (high gelling agents) and agaroids (weak gelling agents) [88,89], depending on the percentages of 3-6-α-l-AnGal*p* residues (B units) and sulfate groups. Agars are essentially isolated from *Gracilaria*, *Gelidium*, or *Pterocladia* algal species, and agarobiose depicts the basic repeating unit of agarose consisting of β-(1→3)-d-Gal*p* units and α-(1→4)-l-An-3,6-*Galp* (Figure 9).

Agars can be considered as mixtures of two polymers (agarose and agaropectin) at variable rates depending on the algal species. From a structural point of view, agarose, with an average M_w_ of 120 kDa (Dp = 400), is often heavily substituted in the C-6 position by methyl groups (until 20%). Contrastingly, agaropectin has the same basic structure as agarose (alternation of residues β-d-Gal*p* and 3,6-An-α-l-Gal*p*) with various substituted groups (high-branching degree), such as glycuronate, methyl, pyruvate, or sulfate [80].

**Figure 10:**
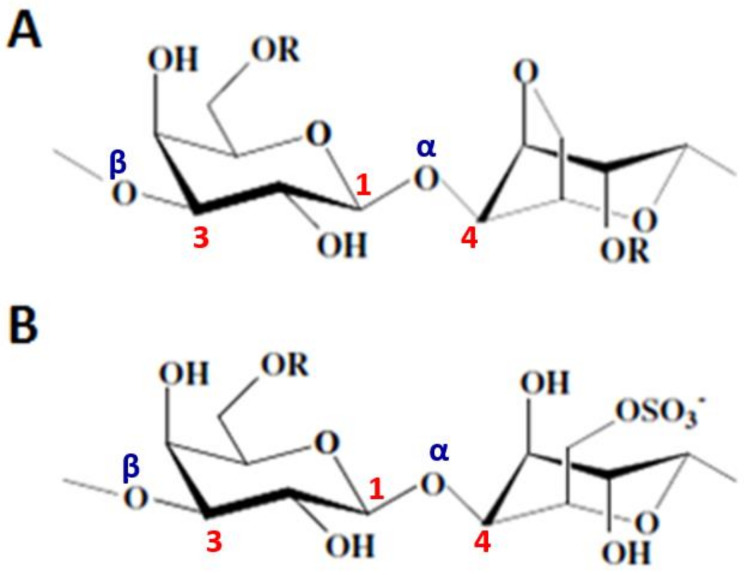
Structures of (**A**) porphyrans and (**B**) funorans. R: CH_3_ or SO_3_^−^.

Agaroids, weakly gelling polymers, have a structure close to agars and can be divided into (i) funorans and (ii) porphyrans (Figure 10). Funorans are constituted of a succession of A (6-*O*-SO_3_^−^) and B (2-*O*-SO_3_^−^) units and are widely used in the field of adhesives. Porphyrans, extracted from *Porphyra* species, e.g., *Porphyra haitanensis* [90,91], *P. capensis* [92], or *P. umbilicalis* [89], have 3-β-d-Gal*p* units methylated or sulfated at C-6 and 50% of B units are 4-α-l-Gal*p*-6-sulfate residues.

### 3.3. Green Seaweed Polysaccharides

**Figure 11:**
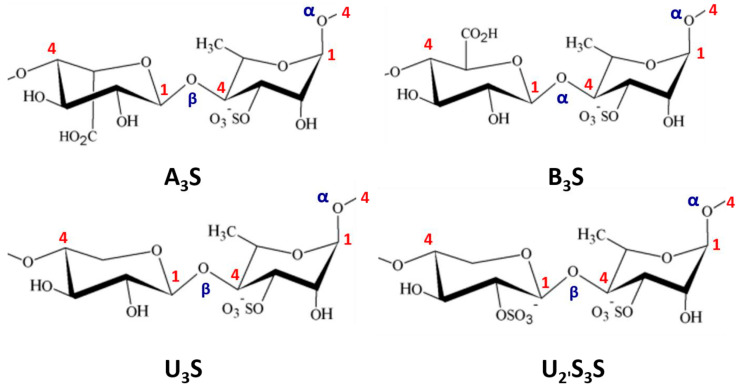
Structures of ulvans. **A_3_S**: ulvanobiuronate-3-sulfate type A,; **B_3_S**: ulvanobiuronate-3-sulfate type B; **U_3_S**: ulvanobiose-3-sulfate type A; **U_2’_S_3_S**: ulvanobiose-2,3-disulfate type B.

Polysaccharides from Chlorophyceae are variously sulfated complex polyholosides that are not fundamentally different from those of terrestrial plants [93]. Their structural patterns can vary depending on the algae species, the culture site and the extraction method. Numerous studies have highlighted three distinct groups [79]. (i) The sulfated xylorhamnoglycuronans, called ulvans, are polyholosides composed of l-Rha*p* (30-50%), d-Xyl*p* (8-9%), d-GlcA*p* (10-20%) and sulfate (16-19%) [94]. The two main recurrent disaccharides in ulvals (*Ulva lactuca*, *U. reticulata*) are β-d-GlcA*p*-(1→4)-α-l-Rha*p*3S-(1→(or A_3_S: ulvanobiuronate-3-sulfate type A) and α-l-IdoA-(1→4)-α-l-Rha*p*3S-(1→ (or B_3_S: ulvanobiuronate-3-sulfate type B) [95] (Figure 11).

Xylose or sulfated xylose (S-Xyl*p*) residues may appear in place of uronic acids in certain polysaccharides. In this case, the disaccharides are respectively composed of ulvanobiose-3-sulfate type A (or U_3_S) and ulvanobiose-2,3-disulfate type B (or U_2’_S_3_S) [96]. Ulvans and their oligomers have been patented for their biological activities that elicit plant defense reactions against biotic and abiotic stresses [97] (Figure 11). (ii) Sulfated xyloarabinogalactans or arabinoxylogalactans (15 to 20% sulfate), present in the orders of Cladophorales and Bryopsidales, are composed of d-Gal*p*, l-Ara*f* and d-Xyl*p* units. These polymers exhibit significant anticoagulant capacities [76]. Finally, (iii) the glucuronoxylorhamnogalactans and the sulfated rhamnogalactogalacturonanes have similar structures to the pectic acids of terrestrial plants and are extracted from some Ulvales [98].

## 4. Health Claims of Algal Polysaccharides

Algal bioactive polysaccharides (ABPs) have been broadly described for their potential human health benefits. Their biological abilities depend on their structural features, e.g., monosaccharidic composition, molecular weight, *O*-glycosidic linkages type (along the polysaccharide backbone and ramifications), sulfation level and position and stereochemistry. Some of these beneficial impacts are discussed and summarized under the following headings.

### 4.1. Antioxidant Activity

**Figure 12:**
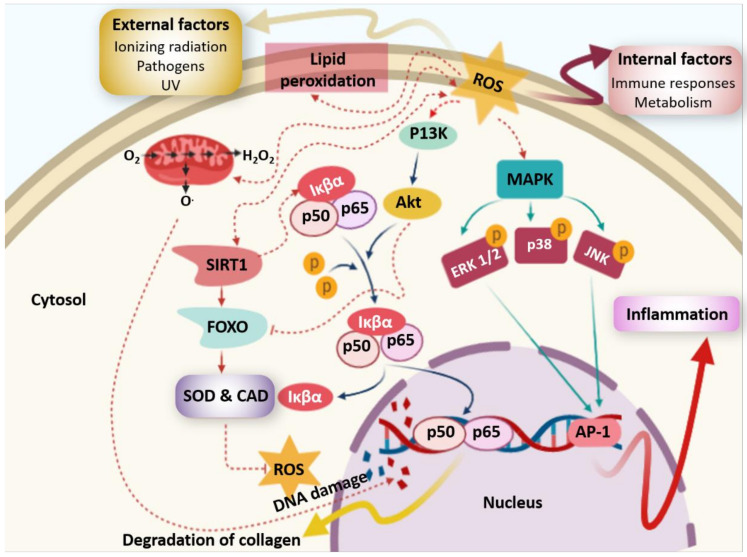
Damage induced by reactive oxygen species (ROS).

Oxidative stress phenomenon is caused by an inequity between production and neutralization of free radicals, which generates various degenerative diseases [9]. Many free radicals, especially reactive oxygen species (ROS), are produced in living organisms over metabolic activities and therefore impact human health (Figure 12).

ROS are produced in the hydrogen peroxide (H_2_O_2_), hydroxyl radical (^•^OH), superoxide radical (O_2_^–^), and nitric oxide (NO) forms. The generated oxidative stress may cause involuntary and pronounced enzyme activation and subsequent oxidative damage to cellular systems. ROS attack and destroy vital macromolecules such as proteins, membrane lipids, and DNA, leading to various disorders such as inflammatory and neurodegenerative diseases, diabetes mellitus, cancer, and intense tissue injuries [71,99,100,101,102] (Figure 12).

In the food industry, butylated hydroxytoluene (BHT), propyl gallate (PG), butylated hydroxyanisole (BHA), and tert-butylhydroquinone (TBHQ) are commonly used as synthetic antioxidants [103]. However, the use of these latter is restricted by food legislation owing to their possible effect in carcinogenesis [85]. For this reason, antioxidant compounds from dietary sources, particularly ABPs, are of cardinal importance for reducing radicals [9]. Antioxidant potential of bioactive molecules has been determined by several methods, such as ABTS (2,2’-azino-bis(3-ethylbenzothiazoline-6-sulfonic acid)) radical scavenging, ferric reducing antioxidant ability (FRAP), DPPH (1,1-diphenyl-2-picrylhydrazyl) radical scavenging, iron (II) chelating activity, nitric oxide scavenging (NO-scavenging), superoxide and hydroxyl radicals scavenging assays, and lipid peroxide inhibition [71,102].

Recently, sulfated polysaccharides (SPs), including fucoidan, sulfated galactan, carrageenan, agar and porphyran isolated from brown and red seaweeds, have been observed to possess appreciable antioxidant abilities (Table 2). According to Choi et al. [104], the sulfated fucans obtained from the brown seaweed *Sargassum fulvellum* are more powerful NO scavengers than commercial antioxidants such as α-tocopherol and BHA. Fucoidans have shown the highest antioxidant activity followed by sodium alginates from brown alga *Cystoseira compressa* according to DPPH and FRAP assays [11]. The chelating, FRAP and anti-DPPH activities of algal SPs depend on the availability of a number of functional groups, such as the carboxylic groups (-COOH) of the d-Man*p*A and l-Gul*p*A units for alginates and the (SO_4_^−^) sulfates groups in the ortho position for fucoidans [11]. Furthermore, it has been mentioned that more than one of -NR_2_, -COOH, -O-, -OH, -C=O, -PO_3_H_2_, -SH, and -S- is in favor of antioxidant capacity [11].

Sudharsan et al. [105] and Li et al. [106] showed that carrageenans and ulvans isolated from Rhodophyceae (*Spyridia hypnoides*) and Chlorophyceae (*Ulva pertusa*), respectively, have great antioxidant properties related to sulfate content. Interestingly, metal chelating and DPPH and ABTS radical scavenging activities of fucan fractions extracted from *Sargassum tenerrimum* appear to relate to low molecular weight and their ratio of sulfate/fucose [107]. Furthermore, the in vivo antioxidant capacity of sulfated galactans derived from Rhodophyceae *Porphyra haitanensis* in aging mice has been signalized [108]. Moreover, according to Li et al. [106], the low molecular weight, uronic acids, and the high sulfate content of ulvans derivatives exhibited improved antioxidant abilities when using the hyperlipidemic Kunming mice model, including glutathione peroxidase, superoxide dismutase, malondialdehyde, and catalase assays in liver. Zhang et al. [108], Costa et al. [109], Souza et al. [110] and Gómez-Ordóñez et al. [111] highlighted the antioxidant effect (in vitro and in vivo) of carrageenans extracted from Rhodophyceae, such as *Mastocarpus stellatus*, *Gracilaria caudata*, *Gracilaria birdiae* and *Porphyra haitanensis*. In another research, De Souza et al. [112] studied the in vitro antioxidant activities of ι-carraghenans (*Eucheuma spinosum*), κ-carrageenans (*Eucheuma cottonii*) and λ-carrageenans (*Gigartina acicularis* and *G. pistillata*), and λ-carrageenan was shown to have the best antioxidant potential compared to κ- and ι-carrageenans. All of these ABPs, especially SPs, could be used as a natural source of antioxidants and are interesting for food cosmetic and pharmaceutical industrial applications.

### 4.2. Anticoagulant and Antithrombotic Activities

Blood coagulation disorders can lead to an increased hazard of clotting (thrombosis) or bleeding (hemorrhage) [113]. As mentioned in the study of Ngo and Kim [114], the blood coagulation mechanism is mediated by coagulation factors, such as IXa, VIIa, Xa, thrombin (FIIa) and VIIIa factors, when an abnormal vascular condition occurs. Exogenous or endogenous anticoagulants can thus interact with the clotting factors to block blood clotting. Heparin, a glycosaminoglycan (highly sulfated polysaccharide) extracted from mammalian tissues (porcine tissue), is currently the commonly and widely anticoagulant/antithrombotic compound used in anticoagulant therapy [115]. However, porcine heparin has been widely criticized for problems with contamination by chondroitin sulfate, which causes hypotension and other undesirable effects (hemorrhagic activity, thrombocytopenia or antithrombin deficiency) [109].

Cui et al. [10] showed that fucoidan fraction NP2 extracted from the Pheophyceae *Nemacystus decipiens* (the Ectocarpales order) can rise the percentage of plasma t-PA/PAI-1 levels, suggesting its high fibrinolytic activity and its possible use as a novel antithrombotic compound. As reported by Zhao et al. [116], the low molecular weight (LMW) fucoidan (M_n_ = 7.3 kDa and M_w_ = 7.6 kDa) isolated from brown alga *Laminaria japonica* exhibited better oral absorption and greater antithrombotic activity, in addition to various antithrombotic mechanisms with regard to those of the middle molecular weight (MMW) fucoidans (M_n_ = 28 kDa and M_w_ = 35 kDa).

Indeed, oral administration of the LMW fucoidan at concentrations of 400 and 800 mg/kg (for 30 days) inhibited the arterial thrombosis formation in rats. It is accompanied by the regulation of TXB2 and 6-keto-PGF1α, significant antiplatelet capacity and efficient fibrinolysis. Anticoagulant properties of fucoidans obtained from brown seaweed *Ecklonia cava* including prothrombin time (PT), thrombin time (TT) and activated partial thromboplastin time (APTT) were recorded [140]. In their further study, Jung et al. [141] endorsed fucoidans isolated from *E. cava* as promising anticoagulants which strongly enhance the inhibition of the ATIII-mediated coagulation factor in coagulation pathways. The study carried out by Wijesinghe et al. [142] demonstrated in vivo anticoagulant activity on Wistar rats of highly sulfated fucoidans extracted from *E. cava*. Nishino and Nagumo [143] and Qui et al. [144] demonstrated that oversulfated fucans exhibited anticoagulant and antithrombin activities and the heparin cofactor II-mediated antithrombin activity of these SPs increased significantly with sulfate content. According to Silva et al. [145], the great sulfation degree at C-3 of (1,4)-α-l-Fuc*p* residues was responsible for the anticoagulant properties of heterofucan isolated from the brown seaweed, *Padina gymnospora*. The sulfated ramified polysaccharide (CP2-1), isolated from the green seaweed *Codium divaricatum*, possessed a high dose-dependent anticoagulant activity evaluated by the APTT, PT and TT assays [146]. Maeda et al. [147] revealed that the anticoagulant-active SPs isolated from *Monostroma nitidum* (Chlorophyceae, Ulotrichales) yielded a six-fold greater capacity than that of heparin. Li et al. [148] and Zhang et al. [139] demonstrated high anticoagulant activities for sulfated rhamnans obtained from *Monostroma latissimum*. Anticoagulant sulfated galactans from the Rhodophyceae *Codium cylindricum* were also characterized by Matsubara et al. [149]. Pereira et al. [150] found that the sulfated galactans from the red alga *Botryocladia occidentalis* have higher anticoagulant properties than similar polysaccharides isolated from *Gelidium crinale* with different sulfation patterns. Sudharsan et al. [151] showed that the sulfated polysaccharide isolated from red marine seaweed *Gracilaria debilis* presented an important anticoagulant ability through APTT and PT (14.11 and 8.23 IU/mg) assays. Silva et al. [145] discussed the anticoagulant properties of sulfated galactans from seaweeds and showed their close dependence on the monosaccharidic composition, the type of glycosidic linkages, the sulfate content, the molar mass and the sulfation position in the backbone structure. Indeed, Fonseca et al. [152] have clearly shown that the differences in venous anticoagulant and antithrombotic activities depend on the yield and the distribution of sulfate groups in the structure of sulfated galactans. In addition, high molecular weight carrageenans with high sulfate content showed higher anticoagulant capacities than those with low sulfate content and a low molecular weight [153]. As reported by Necas and Bartosikova [154], λ-carrageenans have higher antithrombotic activities than those of κ-carrageenans due to their higher sulfate content, but always remain lower than that of heparin.

### 4.3. Anticancer and Antitumor Activities

**Figure 13:**
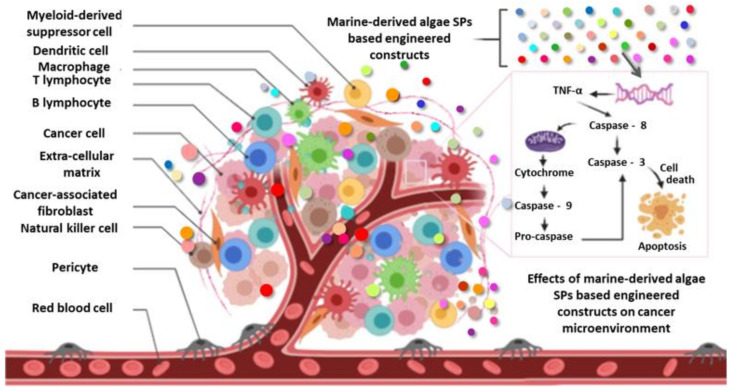
Potentials of marine-derived algae polysaccharide (SP)-based engineered cues to induce cell death of tumor cells (apoptosis).

Today, cancer is considered to be the first causes of human death, and its incidence has doubled in the past decade. This dreadful human disease increases with global warming, unbalanced nutrition, free radicals, and changing lifestyle. The biological mechanism of this pathology involves several families of proteins, such as caspases participating in tumor growth at several stages of carcinogenesis [155]. Therapeutic methodologies using the chemotherapy technique were usually used for the treatment of cancer. However, in most cases, the anticancer agents currently used are particularly cytotoxic. Therefore, many radical scavenging natural compounds, such as SPs from seaweeds, have been proposed for their beneficial effects as cancer prevention agents [124] (Figure 13, Table 2).

Palanisamy et al. [156] reported that fucoidans extracted from *Sargassum polycystum* exhibited antiproliferative properties at a concentration of 50 μg/mL and they can induce apoptosis-mediated cell death against the breast cancer cell line MCF-7 through the activation of caspase-8. According to recent study by Usoltseva et al. [157], the native and deacetylated fucoidans from brown seaweeds *Sargassum feldmannii*, *Sargassum duplicatum*, and their derivatives (at 200 μg/mL) inhibited colony formation of human colon cancer cells (DLD-1, HT-29, and HCT-116). The study findings of Narayani et al. [155] suggest that fucoidan isolated from Brown seaweed *Sargassum cinereum* exerts potent anticancer and apoptotic effects on the human colon adenocarcinoma cell line (Caco-2) by preventing metastasis. As reported by Athukorala et al. [140], SPs isolated from the Pheophyceae *Ecklonia cava* have potential antiproliferative properties on B-16 (mouse melanoma), HL-60 (human promyelocytic leukemia), CT-26 (murine colon carcinoma) and U-937 (human leukemic monocyte lymphoma) cell lines. Porphyran extracted from the red algae *Porphyra yezoensis* can induce in vitro cancer cell death in a dose-dependent manner via apoptosis without affecting normal cells growth [158]. Equally, Souza et al. [129] reported that the kappa-carrageenan obtained from the Rhodophyceae *Hypnea musciformis* (Hm-SP) reduced the proliferation ability on MCF-7 and SH-SY5Y cancer cell-lines, without cytotoxic impact. Chen et al. [159] showed that *Sargassum fusiforme* polysaccharides (SFPS) exhibited a concentration-dependent inhibition of in vitro SPC-A-1 cell proliferation and in vivo tumor growth. All these studies demonstrated that the SFPS administration significantly reduced the tumor microvessel density (MVD) and the expression of CD31, VEGF-A. SFPS also provide the induction of cell cycle arrest and apoptosis of human umbilical vein endothelial cells (HUVECs) and inhibit the VEGF-A expression in tumor cells and its receptor VEGFR2 in HUVECs. Ji and Ji [160] reported the anticancer effect of commercial laminaran (400-1600 μg/mL) on human colon cancer LoVo cells by activating mitochondrial and DR pathways. Moreover, Ji et al. [161] endorsed oversulfated laminaran (1600 μg/mL) to reduce the number of LoVo cells by 86% vs. only 38% for unmodified laminaran at the same concentration. Synytsya et al. [162] suggested that fucoidans extracted from the Korean brown alga *Undaria pinnatifida* have potential antitumor properties in cell lines HepG2 (human hepatocellular liver carcinoma), Hela (human cervical), PC-3 (human prostate), and A549 (carcinomic human alveolar basal epithelial) similar to those of commercial fucoidans. Yan et al. [163] showed that fucoidan obtained from *Sargassum hemiphyllum* can induce miR-29b expression, subsequently helping the inhibition of DNA methyltransferase 3B expression in hepatocellular carcinoma (HCC) human cells. As reported by Lee et al. [164], fucoidans isolated from *Fucus vesiculosus* (Sigma-Aldrich) have been endorsed for their anticancer potentials to induce apoptosis in MC3 human mucoepidermoid carcinoma cells via a caspase-dependent apoptosis signaling cascade (Figure 13).

### 4.4. Immunomodulatory Property

**Figure 14:**
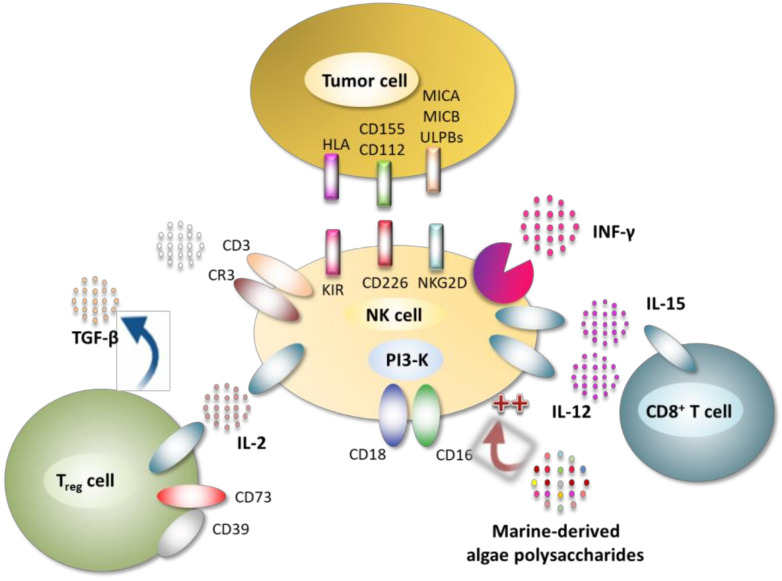
Signaling pathways involved in natural killer cell (NK cells) activation by bioactive algal polysaccharides.

**Figure 15:**
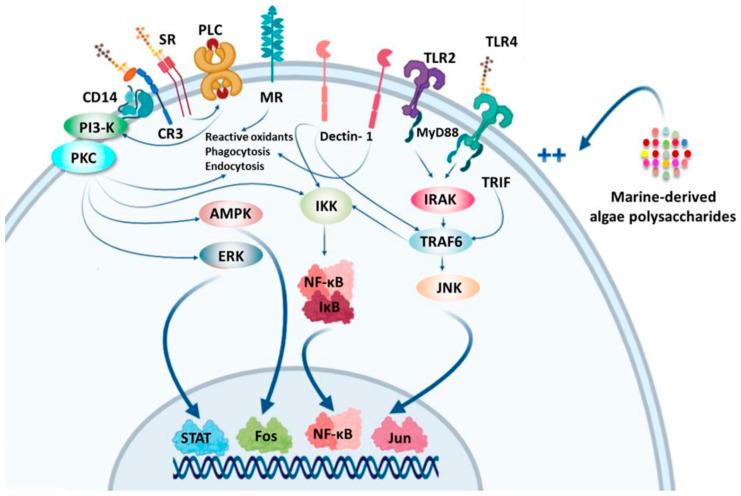
Signaling pathways involved in macrophage activation by algal-sulfated polysaccharides.

Immunomodulation is a therapeutic technique that modulates the balance of cytokines in the human body, either by limiting inflammation and controlling immune reactions, or by stimulating a deficient immune system. Diverse cytokines regulate the activation, development, proliferation, killing of natural killer cells (NK cells) and chemotaxis. According to the study of Raulet [165], NK cell proliferation and the secretion of several cytokines can be stimulated by IL-2 and IL-15. Indeed, the activated NK cells can secrete soluble cytokines such as IFN and TNF to improve the body immune response. SPs derived from algae are biological immunomodulators with an immune regulatory function, and they can maintain homeostasis by regulating NK cells (Figure 14), macrophages (Figure 15), T/B lymphocytes, and complement systems [166].

As biological immunomodulators, fucoidans can activate the immune defense system and inhibit the development of tumor cells by enhancing immunomodulatory activity [167]. Fucoidan can significantly increase the cytolytic activity of NK cells by stimulating macrophage-mediated immune response signaling molecules, such as interleukins IL-2 and IL-12 and IFN-γ [168]. Okai et al. [169] found that fucoidan had stimulating effects on the activity of mouse phagocytic cells (such as B lymphocytes and macrophages) against *Staphylococcus aureus* and on the release of cytokines, IL-1 and TNFα by the same cells. Other studies in mice have also shown significant immunomodulatory effects by increasing the activity of NK cells and modulating the ratio of helper T cells (Th-1/Th-2) [167]. Choi et al. [170] demonstrated the in vitro immunomodulatory effects of fucoidan and arabinogalactan as activators of lymphocytes and macrophages in the immunoprevention of cancer. As reported by Rostami et al. [171], oligoalginates, obtained by enzymatic degradation or physical treatments (high temperature, high pressure or sonication), ensure the stimulation of the proliferation of RAW264.7 macrophages and the production of nitric oxide by these same cells, the growth of human keratinocytes or the growth and migration of endothelial cells. It was reported that five carrageenan types extracted from red seaweeds (belonging to Gigartinaceae and Tichocarpaceae) increased the level of pro-inflammatory IL-6 and TNF-α, and induced the secretion of anti-inflammatory IL-10 in a dose-dependent manner. The differences in activities suggest that the immunomodulatory ability of carrageenans depends on the monosaccharidic composition, the nature of *O*-glycosidic linkages and the number, position and distribution of sulfate groups along the galactan backbone [172]. Bobadilla et al. [173] reported important immunostimulant properties of the soluble (1,3/1,6)-β-d-glucan extracted from brown alga *Durvillaea antarctica*, which induce a 16.9% increase in activated CD19+ B lymphocytes compared with the control sample. Yin et al. [174] suggested that commercial laminarin modulates the immune response and immune-related genes expression in addition to stimulating the growth of the grouper *Epinephelus coioides*. Laminarin supplementation significantly improved the level of total proteins (TP), the lysozyme (LZM), catalase (CAT) and superoxide dismutase (SOD) activities, as well as the expression of immune response genes IL-1β, IL-8, and TLR2 compared with the control. Sulfated polysaccharides purified from the Chlorophyceae *Codium fragile* can ameliorate NK cell activation through the induction of activating receptor expression, cytokine secretion and the release of the perforin, granzyme-B, and lysing proteins [175]. According to Zhao et al. [176], sulfated agarose rich in pyruvate and xylose substitutes, isolated from the red alga *Polysiphonia senticulosa*, displays immunomodulatory activity by improving the viability of NK cells.

### 4.5. Neuroprotective Activity

Neurodegenerative disorders (NDs) are the progressive damage of neurons, mostly concerned with the death of neuronal cells. Numerous types of NDs, such as amyotrophic lateral sclerosis (ALS), Parkinson’s (PD), Alzheimer’s (AD) and prion (PrD) diseases, are associated with the neuronal damage in the different areas of spinal cord and brain [177]. Alzheimer’s disease (AD) is the most common neurodegenerative disorder accounting for 50 to 70% of all dementias [178,179]. AD is mainly characterized by a progressive loss of cognitive functions and memory that could be linked to a significant decrease in brain volume in AD patients when compared to healthy patients, which ultimately leads to disability and dependency [180,181]. Alzheimer’s illness is associated with the failure to clear β amyloid peptide (Aβ) from the walls of blood vessels and plaques in extracellular spaces. The aggregation of Aβ located around neurons has a toxic impact and makes the neurons susceptible to free radicals [179,181]. Currently, this chronic illness afflicts about 20 million people worldwide [182]. Owing to their higher life expectancy and reduction in estrogen levels due to menopause, women are more susceptible to suffer from AD than men [178]. Until now, acetylcholinesterase inhibitors such as donepezil, tacrine, galantamine and rivastigmine are still the most encouraging treatment for AD [183,184,185]. Nevertheless, these inhibitors have a restricted therapeutic success because they merely ameliorate memory in mild dementia and cannot stop the neurodegeneration process [184,186]. Furthermore, these inhibitors have shown several drawbacks in treating AD, such as a narrow therapeutic window, low bioavailability and hepatotoxicity [187]. Therefore, developing novel innocuous drugs to slow down this illness progression is crucial. Recently, Hu et al. [182] reported that fucoidans isolated from ethanol precipitation of *Sargassum fusiform* have therapeutic potential in improving the cognitive dysfunction of mice and enhancing cognitive ability. To delay or halt AD development, this sulfated polysaccharide might be considerably appropriate to treat AD patients. As shown by Wozniak et al. [188], sulfated-fucan extracts obtained by divers from five species of brown seaweeds exhibit antiviral activity against the common herpes simplex virus type 1 (HSV1) in relation to its putative role in AD. The study findings of Luo et al. [189] suggested that fucoidan extracted from *L. japonica* had protective effect on 1-methyl-4-phenyl-1,2,3,6-tetrahydropyridine (a neurotoxin)-induced neurotoxicity in Parkinson’s disease via its antioxidant activity. Considering this scientific evidence, algal-derived polysaccharides can be endorsed for their neuroprotective activities and potential use in clinical therapies. 

## 5. Conclusions

The marine source variety allows for the selection of polysaccharides isolated from seaweeds with specific characteristics, completely absent in polysaccharides from terrestrial plants. Algal polysaccharides and their structural diversity constitute a source of several biological capacities that may represent an interesting tool for novel therapeutic benefits and industrial applications, including cosmeceuticals, nutraceuticals, pharmaceuticals, and functional foods. Currently, sulfated polysaccharides are found principally as excipients in feed, food and pharmaceutical formulations, but the discovery of surprising biological capacities makes these polymers a very exciting research field. For a vision towards the future, the use of algal polysaccharides in medicine is expected to considerably progress. Despite the huge clinical use of alginate gels as wound healing agents, antiulcer and antiacid cures, other bioactive polysaccharides still depict an entirely passive role because of the extraction and purification costs. Thus, the development of novel extraction and purification methodologies of algal polysaccharides might initiate the turning event toward a wide industrial utilization.

## Figures and Tables

**Table 1:** Physicochemical composition of macroalgae.

Macroalgae	Country	Proteins (%)	Lipids (%)	Carbohydrates (%)	Fibers (%)	References
**Brown Seaweeds (Pheophyceae)**						
*Colpomenia sinuosa*	Iran	9.20	1.50	32.10	-	Rohani-Ghadikolaei et al. [33]
*Cystoseira compressa*	Tunisia	9.98	2.80	39.11	57.33	Hentati et al. [34]
*Durvillaea antarctica*	Chile	10.40	0.80	70.90	71.40	Ortiz et al. [35]
*Ecklonia radiata*	New Zealand	9.60	1.80	66.90	-	Smith et al. [36]
*Fucus spiralis*	Portugal	9.71	5.23	17.59	-	Paiva et al. [37]
*Hormosira banksii*	New Zealand	6.07	2.63	62.90	-	Smith et al. [36]
*Padina pavonica*	Iran	11.83	1.79	-	11.00	Tabarsa et al. [38]
*Saccorhiza polyschides*	Portugal	14.44	1.10	45.60	-	Rodrigues et al. [39]
*Sargassum naozhouense*	China	11.20	1.06	47.43	4.83	Peng et al. [40]
**Red Seaweeds (Rhodophyceae)**						
*Ahnfeltia plicata*	Denmark	31.10	1.10	59.10	-	Parjikolaei et al. [41]
*Dumontia contorta*	United Kingdom	31.70	0.12	-	34.30	Marsham et al. [42]
*Gracilaria cervicornis*	Brazil	19.70	0.43	63.10	5.65	Marinho-Soriano et al. [43]
*Jania adhaerens*	Tunisia	9.81	2.76	34.54	51.68	Hentati et al. [34]
*Kappaphycus alvarezii*	India	16.24	0.74	27.40	29.40	Fayaz et al. [44]
*Osmundea pinnatifida*	Portugal	20.79	7.53	17.61	-	Paiva et al. [37]
*Porphyra columbina*	Argentina	24.61	0.25	-	48.02	Cian et al. [45]
**Green Seaweeds (Chlorophyceae)**						
*Caulerpa lentillifera*	Borneo	10.41	1.11	38.66	32.99	Matanjun et al. [46]
*Caulerpa taxifolia*	India	12.44	0.32	23.86	-	Kokilam and Vasuki. [47]
*Ulva lactuca*	Tunisia	8.46	7.87	-	54.90	Yaich et al. [48]
*Ulva reticulata*	Thailand	21.06	0.75	55.77	4.84	Ratana-Arporn and Chirapart [49]
*Ulva rigida*	Spain	17.80	0.90	42.60	11.90	Taboada et al. [50]

**Table 2:** Biological properties of algal bioactive polysaccharides.

Type of PS	Source	Main Monosaccharide	Main Backbone	Biological Properties	References
			**Brown macroalgae**		
S-fucan	*Padina tetrastromatica*	Fuc*p*, Gal*p*, Xyl*p*, Glc*p*Ac	(1,2)- and (1,3)-α-l-Fuc*p*	Nd	Karmakar et al. [117]
S-galactofucans	*Spatoglossum schröederi*	Gal*p*, Fuc*p*, Xyl*p*	(1,4)- and (1,3)-α-l-Fuc*p*	Anti-thrombotic	Costa et al. [109]
S-galactofucans	*Adenocystis utricularis*	Gal*p*, Fuc*p*, Rha*p*, uronic acids	(1,3)-α-l-Fuc*p*	Antiviral	Ponce et al. [69]
S-fucans	*Ascophyllum nodosum*	Fuc*p*, Xyl*p*, Gal*p*, Glc*p*Ac, Glc*p*	(1,3)- and (1,4)-α-l-Fuc*p*	Immunomodulatory, anti-inflammatory, anticoagulant, anti-thrombotic	Cumashi et al. [118]
S-fucans	*Fucus* spp.	Fuc*p*, Xyl*p*, Gal*p*, Glc*p*Ac	(1,3)- and (1,4)-α-l-Fuc*p*	Immunostimulant, antiviral, antitumor, antiproliferative, antiadhesive	Costa et al. [109]
S-galactofucans	*Sargassum* sp.	Gal*p*, Fuc*p*, Rha*p*, Glc*p*Ac	(1,6)-β-d-Gal*p* and (1,2)-β-d-Man*p*	Antitumor	Sokolova et al. [119] Ale et al. [120]
S-fucoidan	*Sargassum horneri*	Fuc*p*	(1,3)-α-l-Fuc*p*, (1,3)- and (1,4)-α-l-Fuc*p*	Antitumor, antiviral	Ale et al. [120] Hoshino et al. [121]
S-fucans	*Ecklonia cava* *Ecklonia kurome*	Fuc*p*, Rha*p*, Gal*p*, Glc*p*Ac	(1,3)- or (1,6)-, and (1,4)-α-l-Fuc*p*	Anti-proliferative, antitumor, anticoagulant, antioxidant, antithrombotic, anti-inflammatory	Ermakova et al. [122] Yamamoto et al. [123]
S-galactofucan	*Laminaria japonica*	Gal*p*, Fuc*p*	(1,3)- and (1,4)-α-l-Fuc*p*	Anti-lipidaemic, antiviral, antitumor, immunomodulator, antioxidant neuroprotective	Fedorov et al. [124] Cumashi et al. [118] Wang et al. [125]
			**Red macroalgae**		
S-λ-carrageenan	*Chondrus crispus*	Gal*p*, AnGal*p*	(1,3)-α-d-Gal*p*, and (1,4)-β-3,6-AnGal*p* or (1,4)-β-d-Gal*p*	Antiviral, anticoagulant, antithrombotic	Albuquerque et al. [126] Luescher-Mattli [127]
S-κ-carrageenan	*E. spinosa*	Gal*p*, AnGal*p*	(1,3)-α-d-Gal*p*, and (1,4)-β-3,6-AnGal*p* or (1,4)-β-d-Gal*p*	Anticoagulant, anti-thrombotic	Prajapati et al. [86] Campo et al. [87]
S-carrageenans	*Stenogramme interrupta*	Gal*p*, AnGal*p*	(1,3)-α-d-Gal*p*, and (1,4)-β-3,6-AnGal*p* or (1,4)-β-d-Gal*p*	Antiviral	Prajapati et al. [86] Caceres et al. [128]
Carrageenan	*Hypnea musciformis*	Gal*p*, AnGal*p*	(1,3)-α-d-Gal*p*, and (1,4)-β-3,6-AnGal*p* or (1,4)-β-d-Gal*p*	Anticancer	Souza et al. [129]
LMW-carrageenans	*Champia feldmannii*	Gal*p*, AnGal*p*	(1,3)-α-d-Gal*p*, and (1,4)-β-3,6-AnGal*p* or (1,4)-β-d-Gal*p*	Antitumor	Lins et al. [130] Prajapati et al. [86] Campo et al. [87]
			**Green macroalgae**		
S-arabinogalactans	*Codium* spp.	Gal*p*, Ara*f*	(1,3)-β-d-Gal	Anticoagulant, antithrombotic, antiviral	Takano et al. [131] Lee et al. [132]
S-ulvans	*Ulva pertusa*	Rha*p*, Xyl*p*, Glc*p*Ac, IdoAc	[→4)-β-d-Glc*p*Ac-(1,4)-α-l-Rha*p*3S-(1→], and [→4)-α-l-IdoAc-(1,4)-α-l-Rha*p*3S-(1→]	Antioxidant, anti-proliferative, hypocholesterolaemic	Usui et al. [133] Xing et al. [134] Yu et al. [135]
S-PS	*Ulva rigida*	Rha*p*, Glc*p*Ac	β-d-Glc*p*Ac-(1,4)-l-Rha*p*	Immunostimulatory	Lahaye and Robic [94] Leiro et al. [136]
S-rhamnans	*Monostroma latissimum*	Rha*p*	(1,3)-α-l-Rha*p*, and (1,3)-α-l-Rha*p* or (1,2)-α-l-Rha*p* or (1→2,3)-α-l-Rha*p*	Antiviral, anticoagulant	Lee et al. [137] Mao et al. [138] Zhang et al. [139]

**PS**: Polysaccharides; **S-PS**: Sulfated polysaccharides; **LMW**: Low Molecular Weight; **Nd**: Not determined.
